# Effect of surgical treatment on patients with stage T3 or T4 triple-negative breast cancer: a SEER-based retrospective observational study

**DOI:** 10.3389/fendo.2023.1184173

**Published:** 2023-05-26

**Authors:** Jie Hu, Changling Dai, Yang Zhang, Weijun Chen, Lihua Sun, Xu Zhang, Minjie Duan, Hao Fu, Teng Long, Wei Kang, Chengliang Yin, Xiaozhu Liu, Jie Yu

**Affiliations:** ^1^ Department of Tumor Radiotherapy, Taizhou Central Hospital (Affiliated Hospital of Taizhou University), Taizhou, China; ^2^ Department of Hematology, Taizhou Central Hospital (Affiliated Hospital of Taizhou University), Taizhou, China; ^3^ College of Medical Informatics, Chongqing Medical University, Chongqing, China; ^4^ School of Biological and Chemical Engineering, Zhejiang University of Science and Technology, Hangzhou, China; ^5^ Department of Infectious Disease, The First Affiliated Hospital of Chongqing Medical University, Chongqing, China; ^6^ Department of Infectious Disease, The Second Affiliated Hospital of Chongqing Medical University, Chongqing, China; ^7^ Department of Mathematics, Physics and Interdisciplinary Studies, Guangzhou Laboratory, Guangzhou, China; ^8^ Faculty of Medicine, Macau University of Science and Technology, Macau, China; ^9^ Department of Cardiology, The Second Affiliated Hospital of Chongqing Medical University, Chongqing, China; ^10^ Department of Medical Imaging, The Affiliated Taian City Central Hospital of Qingdao University, Taian, China

**Keywords:** triple-negative breast cancer, median survival, overall survival, surgical group, non-surgical group

## Abstract

**Background:**

The use of surgery is controversial in patients with stage T3 or T4 triple-negative breast cancer (TNBC). We aimed to explore the effect of surgical treatment on overall survival (OS) of these patients.

**Methods:**

A total of 2,041 patients were selected and divided into the surgical and non-surgical groups based on the Surveillance, Epidemiology, and End Results database from 2010 to 2018. Propensity score matching (PSM) and inverse probability of treatment weighting (IPTW) were applied to balance covariates between different groups. The OS of the two groups were assessed by Kaplan–Meier survival curves and Cox proportional hazards regression models.

**Results:**

A total of 2,041 patients were included in the study. After PSM and IPTW, baseline characteristics of the matched variables were fully balanced. Kaplan–Meier survival curves showed that the median survival time and OS of TNBC patients with stage T3 or T4 in the surgical group were significantly improved compared with those in the non-surgical group. Multivariate Cox proportional hazards regression analysis showed that surgery was a protective factor for prognosis.

**Conclusion:**

Our study found that surgery prolonged the median survival and improved OS compared with the non-surgical group of TNBC patients with stage T3 or T4.

## 1 Introduction

Currently, breast cancer is the most common malignant tumor in women, and its incidence is increasing year by year (1–3). With the increase of women’s life expectancy, a significant number of high-risk tumors have been detected (4, 5). Breast cancer is biologically aggressive and its prognosis is highly variable (6, 7). Triple-negative breast cancer (TNBC) is a highly heterogeneous tumor, accounting for approximately 15% to 20% of all subtypes of breast cancer (8). TNBC is characterized by the lack of expression of estrogen receptor (ER) and progesterone receptor (PR), and no amplification or deletion of human epidermal growth factor receptor 2 (HER2) (9). Compared with other breast cancer subtypes, TNBC patients tend to have higher rates of recurrence, distant metastasis, and lower overall survival (10, 11). Chemotherapy protocols based on anthracyclines and taxanes were considered to be the mainstay of treatment for patients with TNBC because of the lack of hormone receptor targets (12). Previous studies have shown that there are little data exploring the benefits of surgical treatment of patients with stage T3 or 4 TNBC, and the available data have reported controversial results. Several retrospective studies have shown that local surgery is associated with better overall survival in patients with metastatic or specific subtypes of breast cancer (13). However, there was a selection bias in the surgical group. Patients undergoing surgery were generally characterized by younger age, better systemic status, smaller tumors, fewer comorbidities, or lower burden of metastatic disease (14). Nevertheless, two studies, including the prospective phase III trial ABCSG-28 and an open-label randomized controlled trial (RCT), did not find an overall survival benefit in patients with primary stage IV breast cancer after surgical resection (15, 16). The possible reasons for the inconsistent results of RCTS are heterogeneity and selection bias in patients with primary stage IV breast cancer. Therefore, it is important to investigate potential subgroups of patients who may benefit from surgical treatment for primary breast tumors. Even if surgery is not recommended as a routine treatment for all breast cancer patients, selected patients may still benefit.

To date, whether surgery should be the standard of care for patients with TNBC in stage T3 and T4 is a controversial issue. Therefore, the purpose of this study was to evaluate the effect of surgery on overall survival by performing propensity score matching (PSM), inverse probability of treatment weighting (IPTW), multivariate Cox proportional hazards regression analysis, and Kaplan–Meier survival curve for TNBC patients with stage T3 or T4 in the Surveillance, Epidemiology, and End Results (SEER) database. Subgroup analyses were performed to explore population characteristics associated with overall survival.

## 2 Methods

### 2.1 Database and patient characteristics

Patients’ clinical information was extracted from the national cancer database (SEER), which covered approximately 28% of the US population and was grouped by race and ethnicity. The SEER database included patient demographic and clinicopathological variables, first course of treatment, and follow-up data. From 1 January 2010 to 31 December 2018, 18,932 cases diagnosed as TNBC were identified in the SEER data resource. The inclusion criteria are as follows (1): patients who were negative for estrogen, progesterone, and HER2 (2); female; and (3) the TNM stage classification limited to T3 or T4 stage. The exclusion criteria were as follows (1): the primary site was staged as T1 or T2; (2) missing follow-up data; and (3) incomplete clinicopathological information including race, marital status, grade, TNM stage, and therapy.

We conducted a retrospective study of patients with stage T3 or T4 TNBC in the SEER between 2010 and 2018. Demographic and clinicopathological variables included age, race, marital status, tumor grade, location, T stage, N stage, M stage, surgery, radiotherapy, chemotherapy, tumor size, bone metastases, brain metastases, liver metastases, lung metastases, and vital status. About 2,041 patients with stage T3 or T4 TNBC were enrolled in the study. To estimate the effect of surgery on overall survival, the enrolled datasets were stratified into two groups: the surgical and non-surgical. Overall survival, defined as death from any cause, from the date of diagnosis to the last exposure or death, was the primary outcome of the study. Patients who lost the follow-up were reviewed at the last contact. Participants’ consent is not required to access and use SEER data.

### 2.2 Statistical analysis

The numerical variables age and tumor size were summarized as the mean and median [interquartile range (IQR)]. The Shapiro–Wilk test was used to test normality. Categorical variables were presented as counts (%). Demographic and clinicopathological variables were compared between the surgical and non-surgical groups using chi-square test or Fisher’s exact test. The baseline characteristics of the surgical and non-surgical groups were balanced by PSM, and the hazard ratio (HR) was calculated to assess the survival benefit of both groups. Baseline characteristics included age, race, marital status, tumor grade, laterality, N stage, tumor size, radiotherapy, chemotherapy, bone metastases, brain metastases, liver metastases, lung metastases, and other surgery-related factors. A one-to-one (1:1) PSM was conducted to construct a matching sample consisting of pairs of surgical and non-surgical subjects by an optimal matching algorithm to reduce confounding bias. When the absolute value of the mean standardized difference was greater than 0.2 (17), the imbalance is considered unacceptable. The HR and median survival time were calculated to generate Kaplan–Meier survival curves. The log-rank test was employed to compare the survival probability over time between the surgical and non-surgical groups over. IPTW is considered to be a precise method for assessing treatment effects on time-to-event outcome (18). It balanced the baseline variables without losing samples and was used to further reduce the impact of selection bias. Multivariate Cox proportional hazards regression analysis was performed to compare the covariates and explore the relationship between the overall survival rates before and after matching. Proportional hazard assumptions were examined the by Schoenfeld residuals test. Subgroup analyses were performed according to age (≥65 *vs <*65) and stage (T34M0 *vs*. T34M1). Statistical analysis was conducted using R statistical software in version 4.1.2 (www.r-project.org). A two-sided *p*-value < 0.05 was considered statistically significant.

## 3 Results

A total of 2,041 patients with stage T3 or T4 TNBC between 2010 and 2018 were enrolled in this study. As shown in **Table 1**, 413 patients (20.24%) did not receive surgical treatment and 1,628 patients (79.76%) were treated with surgery. Among these patients, the median age was 58, 67.32% were white, 48.41% were married, 60.56% had T3 stage TNBC, 38.90% had N1 stage TNBC, 81.77% had M0 stage, 79.76% had received surgery, 58.26% had received radiotherapy, 80.74% had received chemotherapy, 7.59% had bone metastases, 1.81% had brain metastases, 5.05% had liver metastases, 7.01% had lung metastasis, and 50.76% survived. There were significant differences in age, race, marital status, T stage, N stage, M stage, surgery, radiotherapy, chemotherapy, bone metastases, brain metastases, liver metastases, lung metastases, and status between the non-surgical group and surgical group (*p* < 0.05). Patients who underwent surgery were younger and had longer overall survival than those who did not. Regarding treatment, patients who had surgery were more likely to receive radiation and chemotherapy. Detailed information is shown in **Table 1**.

**Table 1:** The baseline characteristics of 2,041 patients with stage T3 or T4 TNBC from the SEER database.

Characteristic	Overall	Non-surgical	Surgical	*p*-value
	*N* = 2,041	*N* = 413	*N* = 1628	
**Age (median [IQR])**	58.000 [47.000, 69.000]	63.000 [50.000, 73.000]	57.000 [47.000, 68.000]	<0.001
**Race (%)**				<0.001
White	1,374 (67.32)	242 (58.60)	1,132 (69.53)	
Black	421 (20.63)	117 (28.33)	304 (18.67)	
Other	246 (12.05)	54 (13.08)	192 (11.79)	
**Marital status (%)**				<0.001
No	1,053 (51.59)	255 (61.74)	798 (49.02)	
Yes	988 (48.41)	158 (38.26)	830 (50.98)	
**Grade (%)**				0.196
I	13 (0.64)	2 (0.48)	11 (0.68)	
II	318 (15.58)	75 (18.16)	243 (14.93)	
III	1,699 (83.24)	332 (80.39)	1,367 (83.97)	
IV	11 (0.54)	4 (0.97)	7 (0.43)	
**Laterality (%)**				0.282
Left	1,039 (50.91)	200 (48.43)	839 (51.54)	
Right	1,002 (49.09)	213 (51.57)	789 (48.46)	
**T (%)**				<0.001
3	1,236 (60.56)	180 (43.58)	1,056 (64.86)	
4	805 (39.44)	233 (56.42)	572 (35.14)	
**N (%)**				<0.001
0	633 (31.01)	92 (22.28)	541 (33.23)	
1	794 (38.90)	190 (46.00)	604 (37.10)	
2	275 (13.47)	47 (11.38)	228 (14.00)	
3	339 (16.61)	84 (20.34)	255 (15.66)	
**M (%)**				<0.001
0	1,669 (81.77)	201 (48.67)	1,468 (90.17)	
1	372 (18.23)	212 (51.33)	160 (9.83)	
**Surg (%)**				<0.001
No	413 (20.24)	413 (100.00)	0 (0.00)	
Yes	1,628 (79.76)	0 (0.00)	1628 (100.00)	
**Radiation (%)**				<0.001
No	852 (41.74)	313 (75.79)	539 (33.11)	
Yes	1,189 (58.26)	100 (24.21)	1,089 (66.89)	
**Chemotherapy (%)**				<0.001
No	393 (19.26)	136 (32.93)	257 (15.79)	
Yes	1,648 (80.74)	277 (67.07)	1,371 (84.21)	
**Tumor size (median [IQR])**	63.000 [54.000, 82.000]	65.000 [52.000, 90.000]	63.000 [54.000, 80.000]	0.504
**DX bone (%)**				<0.001
No	1,886 (92.41)	314 (76.03)	1,572 (96.56)	
Yes	155 (7.59)	99 (23.97)	56 (3.44)	
**DX brain (%)**				<0.001
No	2,004 (98.19)	384 (92.98)	1,620 (99.51)	
Yes	37 (1.81)	29 (7.02)	8 (0.49)	
**DX liver (%)**				<0.001
No	1,938 (94.95)	345 (83.54)	1,593 (97.85)	
Yes	103 (5.05)	68 (16.46)	35 (2.15)	
**DX lung (%)**				<0.001
No	1,898 (92.99)	326 (78.93)	1,572 (96.56)	
Yes	143 (7.01)	87 (21.07)	56 (3.44)	
**Status (%)**				<0.001
Alive	1,036 (50.76)	129 (31.23)	907 (55.71)	
Dead	1,005 (49.24)	284 (68.77)	721 (44.29)	

TNBC, triple-negative breast cancer.

**Figure 1:**
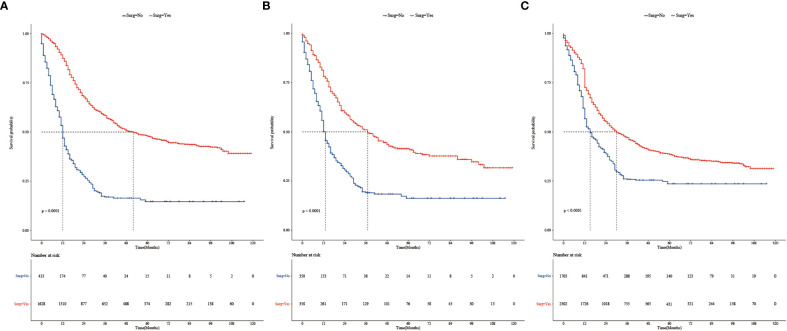
**(A)** Kaplan–Meier survival analysis of 2,041 patients with stage T3 or T4 TNBC in the surgery and non-surgery groups before PSM. **(B)** Kaplan–Meier survival curves of TNBC in the surgery and non-surgery groups after PSM. **(C)** Kaplan–Meier survival curves of patients with TNBC in the surgery and non-surgery groups after IPTW.

To further assess the differences between the surgical and non-surgical groups, one-to-one (1:1) PSM was performed for variables (age, race, marital status, grade, laterality, radiotherapy, chemotherapy, tumor size, N stage, bone metastases, brain metastases, liver metastases, and lung metastases) (**Table 2**). After PSM, the surgical and non-surgical groups consisted of 350 patients respectively. After matching, the baseline characteristics were adequately balanced. Kaplan–Meier curves of the overall survival in the surgical and non-surgical groups before PSM are presented in **Figure 1A**.

**Table 2:** The baseline characteristics of 2,041 patients with stage T3 or T4 TNBC pre- and post-PSM.

	Pre-PSM			Post-PSM		
	Non-surgical	Surgical	*p*	Non-surgical	Surgical	*p*
	*N* = 413	*N* = 1628		*N* = 350	*N* = 350	
**Age (median [IQR])**	63.0 [50.0, 73.0]	57.0 [47.0, 68.0]	<0.001	62.5 [50.0, 73.7]	57.0 [45.0, 72.0]	0.001
**Race (%)**			<0.001			0.001
White	242 (58.60)	1,132 (69.53)		205 (58.57)	247 (70.57)	
Black	117 (28.33)	304 (18.67)		98 (28.00)	51 (14.57)	
Other	54 (13.08)	192 (11.79)		47 (13.43)	52 (14.86)	
**Marital status (%)**			<0.001			<0.001
No	255 (61.74)	798 (49.02)		215 (61.43)	153 (43.71)	
Yes	158 (38.26)	830 (50.98)		135 (38.57)	197 (56.29)	
**Grade (%)**			0.196			0.314
I	2 (0.48)	11 (0.68)		1 (0.29)	5 (1.43)	
II	75 (18.16)	243 (14.93)		65 (18.57)	60 (17.14)	
III	332 (80.39)	1,367 (83.97)		280 (80.00)	283 (80.86)	
IV	4 (0.97)	7 (0.43)		4 (1.14)	2 (0.57)	
**Laterality (%)**			0.282			0.545
Left	200 (48.43)	839 (51.54)		174 (49.71)	183 (52.29)	
Right	213 (51.57)	789 (48.46)		176 (50.29)	167 (47.71)	
**N (%)**			<0.001			0.042
0	92 (22.28)	541 (33.23)		85 (24.29)	117 (33.43)	
1	190 (46.00)	604 (37.10)		160 (45.71)	132 (37.71)	
2	47 (11.38)	228 (14.00)		44 (12.57)	38 (10.86)	
3	84 (20.34)	255 (15.66)		61 (17.43)	63 (18.00)	
**Radiation (%)**			<0.001			<0.001
No	313 (75.79)	539 (33.11)		264 (75.43)	209 (59.71)	
Yes	100 (24.21)	1,089 (66.89)		86 (24.57)	141 (40.29)	
**Chemotherapy (%)**			<0.001			0.033
No	136 (32.93)	257 (15.79)		121 (34.57)	94 (26.86)	
Yes	277 (67.07)	1,371 (84.21)		229 (65.43)	256 (73.14)	
**Tumor size (median [IQR])**	65.0 [52.0, 90.0]	63.0 [54.0, 80.0]	0.504	65.0 [52.0, 86.0]	65.0 [55.0, 80.0]	0.398
**DX bone (%)**			<0.001			0.047
No	314 (76.03)	1,572 (96.56)		293 (83.71)	312 (89.14)	
Yes	99 (23.97)	56 (3.44)		57 (16.29)	38 (10.86)	
**DX brain (%)**			<0.001			0.4961
No	384 (92.98)	1,620 (99.51)		338 (96.57)	342 (97.71)	
Yes	29 (7.02)	8 (0.49)		12 (3.43)	8 (2.29)	
**DX liver (%)**			<0.001			0.1281
No	345 (83.54)	1,593 (97.85)		309 (88.29)	322 (92.00)	
Yes	68 (16.46)	35 (2.15)		41 (11.71)	28 (8.00)	
**DX lung (%)**			<0.001			0.8297
No	326 (78.93)	1,572 (96.56)		301 (86.00)	298 (85.14)	
Yes	87 (21.07)	56 (3.44)		49 (14.00)	52 (14.86)	

PSM, propensity-score matching; TNBC, triple-negative breast cancer.

The survival analysis showed that before PSM, the median survival time was 52 months in the surgical group and 12 months in non-surgical groups. The 3-year survival rate was 58.3% (95% CI: 0.558, 0.610) in the surgical group and 16.9% (95% CI: 0.131, 0.219) in the non-surgical group. The 5-year survival rates were 48.0%, (95% CI: 0.452, 0.509) and 14.6%, (95% CI: 0.107, 0.199), respectively. There were statistically significant difference in median survival time and survival rate between the surgical and non-surgical groups (Log-rank *p* < 0.0001, **Figure 1A**). Surgical treatment significantly improved the median survival time and survival rate of patients with stage T3 or T4 TNBC. After PSM, the median survival time was 37 months and 13 months, respectively (Log-rank *p* < 0.0001, **Figure 1B**). The 3-year survival rate was 51.1% (95% CI: 0.457, 0.571) in the surgical group and 19.0% (95% CI: 0.146, 0.247) in the non-surgical group. The 5-year survival rates were 41.3% (95% CI: 0.359, 0.476) and 16.2% (95% CI: 0.118, 0.223), respectively.

Variables in the raw data were matched using IPTW. The details are shown in **Table 3**. After IPTW, the distributions of most demographic and clinicopathological characteristics were similar between two groups. Kaplan–Meier survival analysis showed that median survival time was 30 months in the surgical group and 15 months in non-surgical groups. Kaplan–Meier curves (**Figure 1C**) showed that the 3- and 5-year overall survival rate in the surgical group (47.1%, 38.6%) were significantly higher than those in the non-surgical groups (25.9%, 23.6%). This is similar to the results of the PSM-matched study cohort.

**Table 3:** The baseline characteristics of patients with TNBC pre- and post-IPTW.

	Pre-IPTW			Post-IPTW		
	No	Yes	*p*	No	Yes	*p*
	*N* = 413	*N* = 1,628		*N* = 1,702.7	*N* = 2,301.75	
**Age (median [IQR])**	63 [50.0, 73.0]	57 [47.0, 68.0]	<0.001	62 [46.012, 73.245]	58 [49.0, 70.0]	0.24
**Race (%)**			<0.001			0.328
White	242 (58.6)	1,132 (69.5)		1,091.1 (64.1)	1,422.0 (61.8)	
Black	117 (28.3)	304 (18.7)		337.8 (19.8)	595.9 (25.9)	
Other	54 (13.1)	192 (11.8)		273.8 (16.1)	283.9 (12.3)	
**Marital status (%)**			<0.001			0.563
No	255 (61.7)	798 (49.0)		875.3 (51.4)	1,251.8 (54.4)	
Yes	158 (38.3)	830 (51.0)		827.4 (48.6)	1,049.9 (45.6)	
**Grade (%)**			0.197			0.025
I	2 (0.5)	11 (0.7)		15.1 (0.9)	14.4 (0.6)	
II	75 (18.2)	243 (14.9)		347.7 (20.4)	298.7 (13.0)	
III	332 (80.4)	1,367 (84.0)		1,318.6 (77.4)	1,980.1 (86.0)	
IV	4 (1.0)	7 (0.4)		21.3 (1.3)	8.6 (0.4)	
**Laterality (%)**			0.282			0.571
Left	200 (48.4)	839 (51.5)		834.1 (49.0)	1,060.5 (46.1)	
Right	213 (51.6)	789 (48.5)		868.6 (51.0)	1,241.3 (53.9)	
**N (%)**			<0.001			0.832
0	92 (22.3)	541 (33.2)		477.3 (28.0)	626.0 (27.2)	
1	190 (46.0)	604 (37.1)		683.3 (40.1)	865.4 (37.6)	
2	47 (11.4)	228 (14.0)		218.1 (12.8)	297.3 (12.9)	
3	84 (20.3)	255 (15.7)		324.0 (19.0)	513.0 (22.3)	
**Radiation (%)**			<0.001			0.547
No	313 (75.8)	539 (33.1)		889.4 (52.2)	1,126.8 (49.0)	
Yes	100 (24.2)	1,089 (66.9)		813.3 (47.8)	1,175.0 (51.0)	
**Chemotherapy (%)**			<0.001			0.053
No	136 (32.9)	257 (15.8)		492.6 (28.9)	495.3 (21.5)	
Yes	277 (67.1)	1,371 (84.2)		1,210.1 (71.1)	1,806.4 (78.5)	
**Tumor size (median [IQR])**	65 [52.0,90.0]	63 [54.0,80.0]	0.5044	65 [52.0,84.0]	65 [55.0,90.0]	0.3345
**DX bone (%)**			<0.001			0.209
No	314 (76.0)	1,572 (96.6)		1,523.3 (89.5)	1,906.5 (82.8)	
Yes	99 (24.0)	56 (3.4)		179.4 (10.5)	395.3 (17.2)	
**DX brain (%)**			<0.001			0.034
No	384 (93.0)	1,620 (99.5)		1,661.2 (97.6)	2,072.5 (90.0)	
Yes	29 (7.0)	8 (0.5)		41.5 (2.4)	229.3 (10.0)	
**DX liver (%)**			<0.001			0.168
No	345 (83.5)	1,593 (97.9)		1,592.4 (93.5)	1,999.5 (86.9)	
Yes	68 (16.5)	35 (2.1)		110.3 (6.5)	302.2 (13.1)	
**DX lung (%)**			<0.001			0.134
No	326 (78.9)	1,572 (96.6)		1,552.3 (91.2)	1,925.4 (83.6)	
Yes	87 (21.1)	56 (3.4)		150.4 (8.8)	376.4 (16.4)	

**Figure 2:**
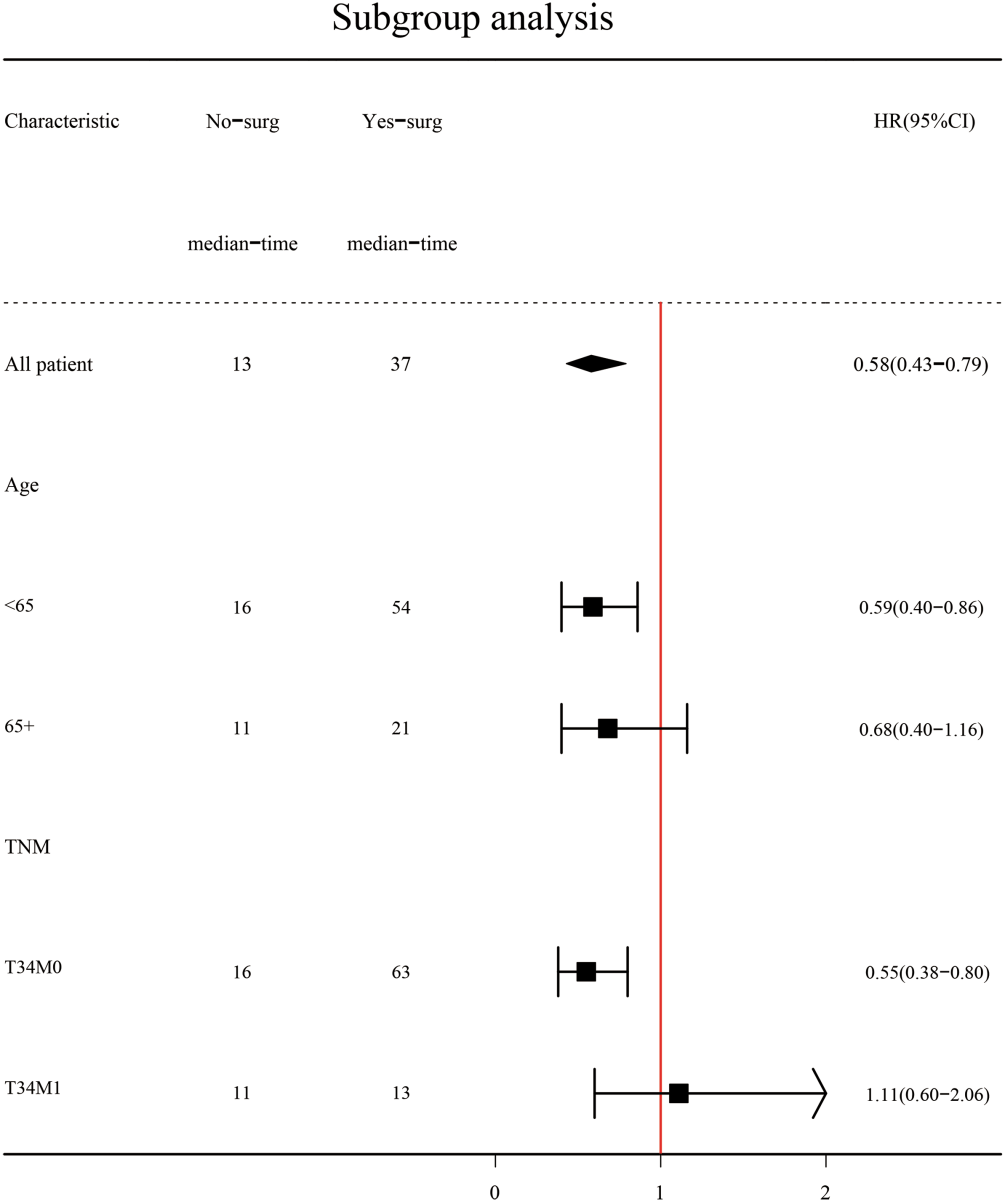
Subgroup analysis after PSM.

**Figure 3:**
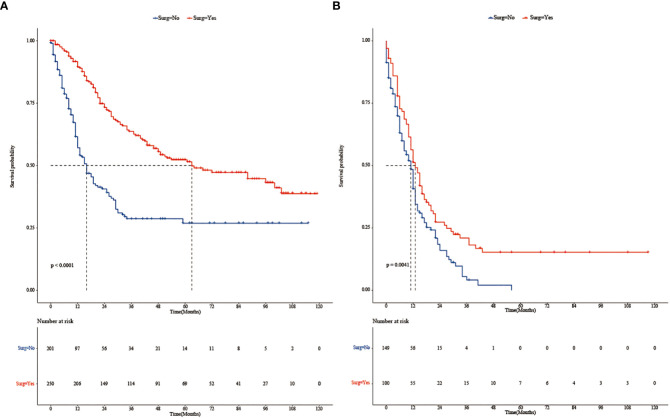
**(A)** Kaplan–Meier survival curves of patients with stage T34M0 TNBC in the surgery and non-surgery groups after PSM. **(B)** Kaplan–Meier survival curves of patients with stage T34M1 TNBC in the surgery and non-surgery groups after PSM.

Subgroup analysis (**Figure 2**) showed that surgery had a significant survival benefit for TNBC patients aged < 65 years in comparison with the non-surgical group (median survival time: 54 *vs*. 16 months) (HR = 0.59, 95% CI: 0.40–0.86). For TNBC patients older than 65 years, there was no significant benefit from surgery (HR: 0.68, 95% CI: 0.4–1.16). Subgroup analysis showed a significant survival benefit (HR: 0.55, 95% CI: 0.38–0.80) for patients with stage T34M0 TNBC. However, there were adverse effects in T34M1 patients receiving surgical treatment (HR: 1.11, 95% CI: 0.60–2.06). After PSM, Kaplan–Meier survival curves of TNBC patients with stage T34M0 (**Figure 3A**) showed that there was a significant difference between the surgical group and the non-surgical group (*p* < 0.001). For TNBC patients with stage T34M1, the Kaplan–Meier survival curves (**Figure 3B**) showed a similar survival benefit in the surgery group (*p* = 0.0041).

Multivariate Cox proportional hazards regression analysis was performed to identify independent predictors of overall survival. Results showed that age, race, marital status, T stage, M stage, N stage, surgery, radiotherapy, chemotherapy, bone metastases, brain metastases, and liver metastases were significantly associated with overall survival (**Table 4**). Age ≥65, T4 stage, N1–3 stage, M1 stage, bone metastases, brain metastases, and liver metastases were associated with poorer overall survival, while race (other), marital status (yes), surgery, radiotherapy, and chemotherapy significantly improved the overall survival (**Table 4**). After PSM, age ≥65 years, T4 stage, N1–3 stage, M1 stage, bone metastases, and liver metastases were associated with lower overall survival, while races (other: not white or black), surgery, and chemotherapy improved overall survival (**Table 4**).

**Table 4:** Multivariate Cox proportional hazards regression analysis before PSM and after PSM.

Characteristic	Before PSM		After PSM	
	HR (95% CI)	*p*	HR (95% CI)	*p*
**Age**				
<65				
65+	1.31 (1.14–1.5)	<0.001	1.58 (1.26–1.98)	<0.001
**Race**				
White				
Black	1.07 (0.91–1.25)	0.4207	0.92 (0.71–1.2)	0.55
Other	0.8 (0.65–0.98)	0.0324	0.65 (0.49–0.88)	<0.001
**Marital status**				
No				
Yes	0.83 (0.73–0.95)	0.0064	0.93 (0.75–1.16)	0.52
**Grade**				
I				
II	1.22 (0.5–3)	0.6588	1.1 (0.34–3.52)	0.8737
III	1.29 (0.53–3.12)	0.5739	1.14 (0.36–3.58)	0.8274
IV	0.86 (0.24–3)	0.8097	1.02 (0.22–4.76)	0.9784
**Laterality**				
Left				
Right	1 (0.88–1.13)	0.9841	0.98 (0.8–1.19)	0.8326
**T**				
3				
4	1.37 (1.2–1.56)	<0.001	1.4 (1.14–1.73)	0.0016
**N**				
0				
1	1.37 (1.16–1.63)	<0.001	1.33 (1.01–1.75)	0.0427
2	2.05 (1.67–2.51)	<0.001	1.87 (1.33–2.64)	<0.001
3	2.18 (1.79–2.67)	<0.001	1.8 (1.3–2.49)	<0.001
**M**				
0				
1	1.56 (1.22–1.99)	<0.001	1.77 (1.25–2.5)	0.0012
**Surg**				
No				
Yes	0.55 (0.47–0.66)	<0.001	0.54 (0.44–0.67)	<0.001
**Radiation**				
No				
Yes	0.76 (0.66–0.88)	<0.001	0.95 (0.76–1.18)	0.6399
**Chemotherapy**				
No				
Yes	0.47 (0.4–0.55)	<0.001	0.46 (0.36–0.59)	<0.001
**Tumor size**	1 (1–1.02)	0.3412	1 (1–1.02)	0.2983
**DX bone**				
No				
Yes	1.5 (1.17–1.92)	0.0016	1.58 (1.15–2.17)	0.0051
**DX brain**				
No				
Yes	2.09 (1.41–3.1)	<0.001	1.51 (0.87–2.63)	0.1438
**DX liver**				
No				
Yes	1.92 (1.48–2.48)	<0.001	2.09 (1.52–2.88)	<0.001
**DX lung**				
No				
Yes	1.21 (0.94–1.57)	0.1406	1.14 (0.82–1.59)	0.434

## 4 Discussion

Multiple retrospective studies have revealed the potential benefits of surgery for advanced breast cancer (19, 20). A study based on the National Cancer Database (NCDB) highlighted that surgery can benefit patients with stage IV breast cancer. In this large cohort (11,694), even after PSM, the overall survival of the surgical group was improved compared with the non-surgical group (HR = 0.68, 95% CI: 0.63–0.72, *p* < 0.001) (13). This is because surgery may substantially reduce the overall tumor burden and improve survival by activating the immune responsiveness (21). However, other studies held different opinions. TATA, TBCRC013, and positive clinical trials suggested that surgical treatment had a similar prognosis for patients with stage IV breast cancer compared with the non-surgical treatment (15, 16, 22). Xie et al. found that most patients with metastatic breast cancer have benefited from local regional primary tumor surgery. However, the PSM dataset showed that surgery did not prolong the breast cancer-specific survival of TNBC patients. At present, there are few studies on the surgical treatment of TNBC patients with stage T3 or T4, and there is no clear clinical consensus. Therefore, it is important to study the potential subgroup of patients with favorable features for surgical treatment of the primary breast tumors. We had investigated whether there was a survival benefit from surgical treatment in patients with stage T3 or T4 TNBC.

In this cohort study, we sought to reveal distinct outcomes of patients with or without surgical treatment for TNBC at stage T3 or T4, based on population data from SEER. To our knowledge, this is the first study to compare the impact of surgery on patients with stage T3 or 4 TNBC. We found that TNBC patients with stage T3 or T4 had a higher proportion of surgery and better overall survival than those who did not. Before PSM, the median survival time, 3-year overall survival rate, and 5-year overall survival rate of patients were significantly higher in the surgical group than those in the non-surgical group. Log-rank test of the Kaplan–Meier curve revealed that there were significant differences in the median survival time and overall survival between two groups (*p* < 0.001). Surgery significantly improved overall survival in the study population compared with the non-surgical group. In order to reduce the bias caused by confounding factors, PSM was conducted. Kaplan–Meier curves still showed the same results after PSM when baseline characteristics of the surgical and non-surgical groups were completely balanced (*p* < 0.001). We matched the variables in the original data with IPTW, resulting in a similar distribution of most demographic and clinicopathological characteristics between the two groups. We found that surgery significantly improved overall survival definitely.

To explore the effect of surgery on overall survival among TNBC patients with different ages (<65 *vs*. ≥65) and stages (T34M0 *vs*. T34M1), we performed subgroup analyses. We found that for TNBC patients aged < 65 years, the median survival time was significantly longer in the surgical group than in the non-surgical group. It may be caused by the small sample size, the HR was 0.68 (95% CI: 0.4–1.16) of TNBC patients aged >65 years, and the HR value was 1.11 (95% CI: 0.60–2.06) of TNBC patients at stage T34M1. Subgroup analysis showed that TNBC patients at stage T34M0 had a significant survival benefit after surgery. After matching, Kaplan–Meier survival curves of patients with stage T34M0 TNBC showed statistical difference between two groups (*p* < 0.001), and surgery had significant survival benefit. After matching, Kaplan–Meier survival curves of the two groups of TNBC patients at stage T34M1 was statistically different (*p* = 0.0041), and there was survival benefit from surgical treatment. The two curves were close. In multivariate Cox proportional hazards regression analysis, we found that surgery was an independent predictor of overall survival. This is consistent with the results of a propensity score matching analysis based on the SEER population (23). However, Li et al. proposed that surgical resection could reduce the burden of the local tumor, thereby improving the overall survival rate. For patients with visceral or multiple metastases, local lesion size and number of lymph node metastasis had little influence on systemic tumor burden, and local surgery had limited impact (24). Our study found that for patients with distant metastasis, surgery can reduce tumor burden, control local symptoms, and improve quality, but individualized treatment is required.

However, several limitations should also be mentioned in this study. Firstly, the selection bias regarding the retrospective design remains even when PSM and IPTW were utilized. Secondly, the status of disease burden is incomplete (the SEER database does not provide information on the number of metastases, only on major sites, such as bone, lung, liver, brain, and distant lymph nodes). We were unable to control for these potential modifiers. Finally, we were unable to assess recurrence because SEER does not collect this information. Despite these limitations, our study, which used population-based data, provided valuable new information on the effectiveness of surgical treatment in patients with stage T3 or T4 TNBC.

## 5 Conclusion

Our study found that surgical treatment prolonged median survival and improved overall survival of patients with stage T3 or T4 TNBC compared with the non-surgical group in patients. Nevertheless, these findings need to be further validated and explored in future large-scale observational clinical studies.

## Data availability statement

The original contributions presented in the study are included in the article/supplementary material. Further inquiries can be directed to the corresponding authors.

## Author contributions

JY and XL contributed to the conception and design. CD, YZ, WC, LS, XZ, MD, HF, TL, and WK analyzed the data. JH drafted the manuscript. CY, XL, and JY contributed to the critical revision of the manuscript. All authors contributed to the article and approved the submitted version.
